# Digital Papillary Adenocarcinoma Is HPV-42-Associated and BRAFV600E Negative: Perspectives for Diagnostic Practice

**DOI:** 10.3390/dermatopathology11040037

**Published:** 2024-12-09

**Authors:** Tassilo Dege, Arno Rütten, Matthias Goebeler, Hermann Kneitz

**Affiliations:** 1 Department of Dermatology, Venereology and Allergology, University Hospital Würzburg, 97080 Würzburg, Germany; 2 Dermatopathology Friedrichshafen, 88048 Friedrichshafen, Germany

**Keywords:** DPAC, HPV, HPV-42, oncogenesis, adnexal tumor, digital papillary adenocarcinoma, molecular pathology, BRAF, p16

## Abstract

Digital papillary adenocarcinoma (DPAC) is a rare, low-grade sweat gland carcinoma primarily found on the hands, fingers, or toes and predominantly affecting males. Distinguishing DPAC from benign sweat gland tumors can be challenging. We present the case of a 52-year-old patient with a progressive tumor on the finger initially misdiagnosed as a viral wart. Histological examination revealed a cytologically basophilic sweat gland tumor with tubular structures, papillary protrusions, and a characteristic immunohistochemical staining pattern for CK 7 and Actin. HPV-42 positivity and molecular analysis confirmed the diagnosis of DPAC. HPV-42 has been strongly associated with DPAC. Additionally, p16 positivity and BRAFV600E negativity were observed. These findings aid in the differential diagnosis of acral sweat gland tumors and guide clinical management, including with respect to the potential for recurrence and metastasis.

## 1. Case Presentation

**Figure 1:**
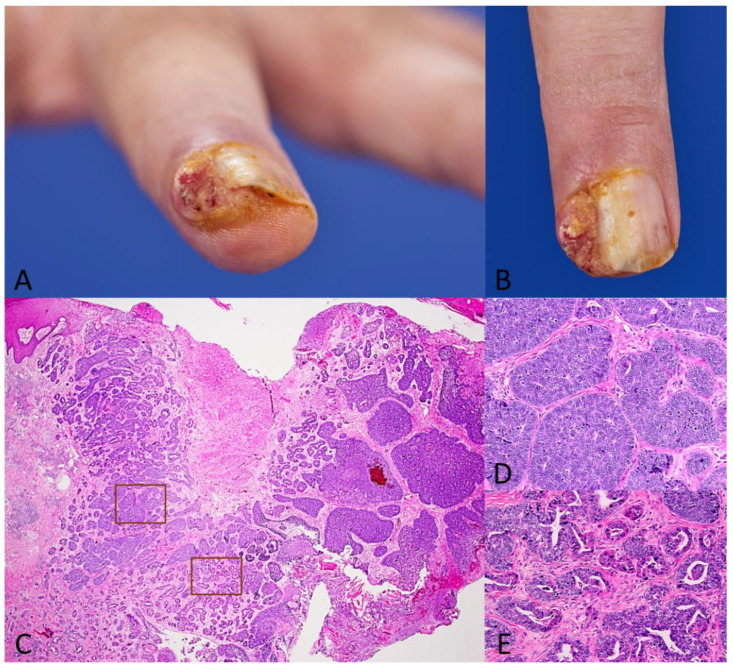
(**A**,**B**): Left middle finger distal phalanx with a centrally ulcerated, skin-colored tumor measuring 1.8 cm × 1.1 cm. (**C**–**E**): Hemotoxylin–Eosin stain showing an adnexal tumor (**C**, overview, 25×) with solid proliferations of pleomorphic, basophilic cells (**D**, 40×) and tubular structures with signs of decapitation secretion (**E**, 40×).

A 52-year-old patient developed a progressive and painful tumor on his left middle finger which he first noticed a year before (Figure 1A,B). External curettages were performed multiple times under the suspected diagnosis of a viral wart (and therefore no histological examination was conducted), but the tumor kept recurring. Eventually, there was significant growth with ulceration of the tumor, leading to an external biopsy, which was evaluated at our facility. Histologically, there was suspicion of a malignant adnexal tumor, prompting us to perform a micrographic surgery (with a 5 mm safety margin) on the tumor at our department. Sonography of the axillary lymph nodes and computed tomography of the thorax and the abdomen were performed to rule out distant metastasis.

Histological examination revealed a poorly demarcated, ulcerated (most likely due to previous curettages), and cytologically basophilic-appearing sweat gland tumor without any connection to the epidermis (Figure 1C). Besides solid proliferations with moderately pleomorphic, basophilic cell growth (Figure 1D), numerous tubular structures with signs of decapitation secretion were evident (Figure 1E). Within tubular and adenoid structures, a partially multi-layered epithelium on which papillary protrusions extending into the lumen had formed was conspicuous.

Immunohistochemistry was performed using antibodies directed against HPV (DCS, monoclonal, K1 H8), p16 (Medac, monoclonal, Clone MX007), BRAFV600E (abcam, monoclonal, Clone VE 1), CK7 (DAKO, monoclonal, Clone OV-TL 12/30), Actin (Cellmarque, monoclonal, Clone HHF35), and Ki-67 (Dako, monoclonal, Clone MIB-1) as described. Polymerase chain reactions (PCRs) were used for the amplification, identification, and characterization of Human Papillomavirus type 42 (HPV-42).

**Figure 2:**
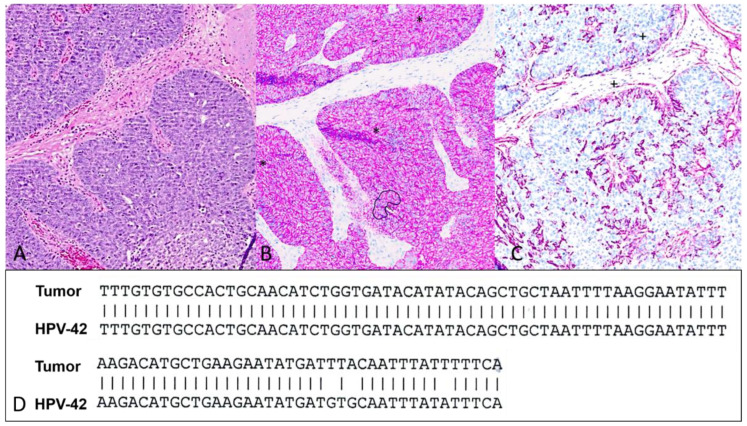
(**A**–**C**): Hemotoxylin–Eosin stain (**A**, 40×), correlated with immunostains, showing tumor cell complexes with heterogeneous expression of CK7 (*) (**B**, 40×). An outer actin-positive (+) myoepithelial layer (**C**, 40×) surrounds tumor cells. (**D**): Sequence of tumor DNA matching HPV-42.

**Figure 3:**
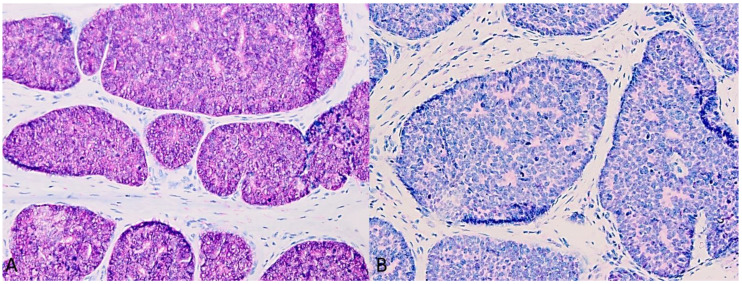
(**A**): Tumor cells stain strongly positive for p16. (**B**): Tumor cells immunohistochemically negative for BRAFV600E.

The tumor cell complexes (Figure 2A) showed a heterogeneous expression of CK7 (Figure 2B), and the cell aggregates were predominantly surrounded by an outer actin-positive myoepithelial layer throughout (Figure 2C). This characteristic immunohistochemical staining pattern for CK7 and Actin serves as an immunohistochemical clue. Tumor cells showed increased immunolabelling with Ki-67. Immunostainings were positive for p16 (Figure 3A) but negative for BRAFV600E (Figure 3B). Moreover, PCR and sequencing were performed, allowing the detection of HPV-42 DNA (Figure 2D). After summarizing all the findings, we made a diagnosis of digital papillary adenocarcinoma (DPAC). During the follow-up (currently > 2 years), neither tumor recurrence nor metastasis was observed.

## 2. Discussion

DPAC is a cutaneous sweat gland malignancy with high potential for aggressive local invasion. It is predominantly localized at the hands, fingers, or toes, with very rare exceptions. It was first studied in a larger case series in 1987 [1] and later referred to as aggressive digital papillary adenocarcinoma. DPAC is rare (incidence: 0.08/1,000,000), affects mainly males (male-to-female ratio: 4:1), tends to locally recur, and may metastasize up to even 20 years after an initial diagnosis [2]. A unique presentation of DPAC with multiple cutaneous nodules and a verrucous plaque arranged in a sporotrichoid distribution on an upper limb, mimicking an infectious condition, has also been described [3]. Clinically, it can manifest as nail bed infection (paronychia), be misinterpreted as a synovial cyst, or mimic a giant-cell tumor [4,5,6].

The recommended treatment approach for DPAC is wide excision or digital amputation, with or without sentinel lymph node biopsy, followed by long-term surveillance. Micrographic surgery offers the advantage of ensuring histologic margin clearance and functional preservation. Recently, cutaneous adnexal carcinomas including DPAC have been found to express Nectin-4 [7]; the anti-nectin-4 antibody-drug conjugate enfortunab-vedotin, recently FDA- and EMA-approved for locally advanced or metastasized bladder carcinoma [8], may have enough potential that is should be considered for advanced DPAC.

DPAC exhibits a wide histomorphological spectrum, ranging from a hidradenoma- or cystadenoma-like appearance to highly pleomorphic infiltrative epithelial proliferations with evident cellular and nuclear atypia [9]. Histologically, DPAC typically manifests as a poorly circumscribed, multilobular lesion. It is composed of well-formed atypical glands with papillary projections, which are lined by malignant cells with moderate atypia and few mitoses. The solid component includes tubuloalveolar and ductal structures with areas of papillary projections protruding into cystic lumina. These solid structures are lined by cuboidal to columnar CK 7 positive epithelia that are surrounded by an actin-positive basal myoepithelial layer. It has been suggested by Suchak et al. that the presence of tumor-associated myoepithelial cells should not be interpreted as benign but rather prompt the need for clinical or histopathological evaluation to determine the primary adnexal origin of the tumor [2]. The glandular lumina may contain eosinophilic secretory material. Cytologic atypia is generally mild to moderate, and scattered mitotic figures are present. The parenchymal component shows variation in appearance and may range from thin fibrous to dense hyalinized collagen. In some cases, there may be focal necrosis, lymphatic invasion, or infiltration of underlying soft tissue and bone.

The pathogenesis of DPAC was recently associated with HPV-42. Vanderbilt et al. showed a strong association between the presence of HPV-42 and DPAC [10]. All eight DPAC cases examined showed positivity for HPV-42 (8/8), whereas all other acral hidradenomas or sweat gland tumors tested were negative for this virus (0/22). Moreover, the presence of the HPV-42 genome and the expression of its viral oncogenes have been confirmed in non-acral DPAC (5/5) [11]. Leiendecker et al. identified HPV-42 in 96% of the DPAC cases they examined (45/47) and demonstrated the integration of viral oncogenes such as *E6* and *E7* in most DPAC cases. HPV-42, previously regarded as a “low-risk” virus, is now considered a driver of DPAC development. However, the presence of BRAF mutations was not tested in these tumors. While prophylactic HPV vaccination effectively prevents HPV-associated diseases, current vaccines target only the most prevalent HPV types [12] (the most common HPV types safeguarded against are 6, 11, 16, and 18, and the 9-valent vaccine additionally contains HPV types 31, 33, 45, 52, and 58). This supports the inclusion of additional HPV types in the development of new vaccines, emphasizing the potential of pan-HPV vaccines [13].

Based on these recent findings [14], it can be presumed that the additional detection of HPV-42 indicates the presence of DPAC and can be used in the differential diagnosis of other acral sweat gland tumors. In the case presented here, molecular pathological analysis also revealed positivity for HPV-42. Despite the tumor having a relatively small cystic component, the immunohistochemical results support the diagnosis of DPAC [15]. However, HPV-42 is not present in all cases of DPAC, so pathologists should not rely solely on HPV-42 when differentiating DPAC from other papillary adnexal neoplasms [16].

Histological differential diagnosis of acral adnexal tumors, particularly distinguishing DPAC from acral hidradenomas, acral poroma, myoepithelioma, papillary eccrine adenoma (PEA), tubular apocrine adenoma, and cystadenoma, is often challenging in diagnostic practice, often leading to delayed treatment owing to misdiagnosis [17].

One of DPAC’s most distinguishing features is the presence of papillary structures, both micropapillae and macropapillae, which are almost invariably present in digital papillary adenocarcinoma but typically absent in acral hidradenoma [18]. As an important differential diagnosis, PEA is a rare and benign adnexal tumor characterized by well-circumscribed, dilated ducts with micropapillae. PEA lacks large cysts, solid areas, significant nuclear atypia, or notable mitotic activity [19,20] and harbors a BRAFV600E mutation [21,22]. When one suspects DPAC and notes the presence of a BRAFV600E mutation, PEA should always be carefully considered before diagnosing a BRAFV600E-mutated DPAC.

## 3. Conclusions

The recently discovered strong association between HPV-42 and DPAC can aid in diagnosing DPAC in difficult cases. This has significant clinical importance for affected patients, as DPAC exhibits a markedly increased tendency for recurrence compared to other acral sweat gland tumors and, in rare cases, can also metastasize [23]. Clinically, immunohistochemical and molecular markers like HPV-42, p16 (as a surrogate), and BRAFV600E can guide clinicians in differentiating between DPAC and its differential diagnoses so that additional diagnostics (i.e., a sentinel lymph node biopsy) and aftercare can be provided in a more personalized manner [24].

## Data Availability

Data are contained within this article.

## References

[1] Kao G.F., Helwig E.B., Graham J.H. (1987). Aggressive digital papillary adenoma and adenocarcinoma. A clinicopathological study of 57 patients, with histochemical, immunopathological, and ultrastructural observations. J. Cutan. Pathol..

[2] Suchak R., Wang W.L., Prieto V.G., Ivan D., Lazar A.J., Brenn T., Calonje E. (2012). Cutaneous digital papillary adenocarcinoma: A clinicopathologic study of 31 cases of a rare neoplasm with new observations. Am. J. Surg. Pathol..

[3] Gupta A., Khullar G., Divyashree R., Sharma S. (2024). Unusual presentation of digital papillary adenocarcinoma in a sporotrichoid pattern. Indian J. Dermatol. Venereol. Leprol..

[4] Gorva A.D., Mohil R., Srinivasan M.S. (2005). Aggressive digital papillary adenocarcinoma presenting as a paronychia of the finger. J. Hand Surg. Br..

[5] Martínez Villén G., Alvarez Alegret R., Canales V., Herrera A. (2012). Aggressive digital papillary adenocarcinoma of the hand: An unsuspected malignant tumour of the sweat glands. J. Plast. Surg. Hand Surg..

[6] Joyce K., Leonard N., Theopold C. (2020). Aggressive Digital Papillary Adenocarcinoma Mimicking a Giant Cell Tumour—A Case Report and Review of the Literature. Cureus.

[7] Cho W.C., Saade R., Nagarajan P., Aung P.P., Milton D.R., Marques-Piubelli M.L., Hudgens C., Ledesma D., Nelson K., Ivan D. (2024). Nectin-4 expression in a subset of cutaneous adnexal carcinomas: A potential target for therapy with enfortumab vedotin. J. Cutan. Pathol..

[8] Wong J.L., Rosenberg J.E. (2021). Targeting nectin-4 by antibody-drug conjugates for the treatment of urothelial carcinoma. Expert Opin. Biol. Ther..

[9] Held L., Mentzel T., Paredes B., Griewank K., Itzlinger-Monshi B., Rütten A. (2019). Digital papillary adenocarcinoma: Four case reports with brief literature review. Der. Hautarzt..

[10] Vanderbilt C., Brenn T., Moy A.P., Harloe G., Ariyan C., Athanasian E., Busam K.J. (2022). Association of HPV42 with digital papillary adenocarcinoma and the use of in situ hybridization for its distinction from acral hidradenoma and diagnosis at non-acral sites. Mod. Pathol..

[11] Kervarrec T., Imbeaud S., Veyer D., Pere H., Puech J., Pekár-Lukacs A., Markiewicz D., Coutts M., Tallet A., Collin C. (2023). Digital Papillary Adenocarcinoma in Nonacral Skin: Clinicopathologic and Genetic Characterization of 5 Cases. Am. J. Surg. Pathol..

[12] Falcaro M., Castañon A., Ndlela B., Checchi M., Soldan K., Lopez-Bernal J., Elliss-Brookes L., Sasieni P. (2021). The effects of the national HPV vaccination programme in England, UK, on cervical cancer and grade 3 cervical intraepithelial neoplasia incidence: A register-based observational study. Lancet.

[13] Olczak P., Matsui K., Wong M., Alvarez J., Lambert P., Christensen N.D., Hu J., Huber B., Kirnbauer R., Wang J.W. (2022). RG2-VLP: A vaccine designed to broadly protect against anogenital and skin human papillomaviruses causing human cancer. J. Virol..

[14] Bui C.M., Pukhalskaya T., Smoller B.R., Zengin H.B., Heneidi S., Vail E., Makhoul E., Balzer B. (2023). Two distinct pathogenic pathways of digital papillary adenocarcinoma—BRAF mutation or low-risk HPV infection. J. Cutan. Pathol..

[15] Duke W.H., Sherrod T.T., Lupton G.P. (2000). Aggressive digital papillary adenocarcinoma: (Aggressive digital papillary adenoma and adenocarcinoma revisited). Am. J. Surg. Pathol..

[16] Chen F., Nagarajan P., Aung P.P. (2024). Digital Papillary Adenocarcinoma: The Detection of Low-Risk Human Papillomaviruses and the BRAF p.V600E Mutation in a Subset of Cases. Dermatopathology.

[17] Dhawan S.S., Nanda V.S., Grekin S., Rabinovitz H.S. (1990). Apocrine adenocarcinoma: Case report and review of the literature. J. Dermatol. Surg. Oncol..

[18] Wiedemeyer K., Gill P., Schneider M., Kind P., Brenn T. (2020). Clinicopathologic characterization of hidradenoma on acral sites: A diagnostic pitfall with digital papillary adenocarcinoma. Am. J. Surg. Pathol..

[19] Galadari E., Mehregan A., Lee K. (1987). Malignant transformation of eccrine tumors. J. Cutan. Pathol..

[20] Scolyer R.A., Karim R.Z., Thompson J.F., Stretch J.R., McCarthy S.W., Murali R. (2013). Digital papillary adenocarcinoma: A tumour that should be considered in the differential diagnosis of neoplasms involving the digits. Pathology.

[21] Liau J.-Y., Tsai J.-H., Huang W.-C., Lan J., Hong J.-B., Yuan C.-T. (2018). BRAF and KRAS mutations in tubular apocrine adenoma and papillary eccrine adenoma of the skin. Hum. Pathol..

[22] Nguyen A.J., Johnson E., Camilleri M., Wieland C., Lehman J.S., Agrawal S., Comfere N., Fadra N., Knudson R.A., Greipp P. (2024). Ancillary immunohistochemical and molecular testing in the classification of cutaneous sweat gland/duct neoplasms: A validation study with emphasis on histomorphologic correlation and pathological diagnosis. Hum. Pathol..

[23] Chen S., Asgari M. (2014). Is aggressive digital papillary adenocarcinoma really aggressive digital papillary adenocarcinoma?. Dermatol. Pract. Concept..

[24] Kervarrec T., Busam K.J. (2023). Acral BRAF-mutated tubular adenoma should be distinguished from HPV42-related digital papillary adenocarcinoma. J. Cutan. Pathol..

